# Functional Settings of Hospital Outdoor Spaces and the Perceptions from Public and Hospital Occupant during COVID-19

**DOI:** 10.3390/healthcare9111534

**Published:** 2021-11-10

**Authors:** Ming Ma, Michael Adeney, Hao Long

**Affiliations:** 1 Collage of Architecture and Urban Planning, Chongqing University, Chongqing 400030, China; Longhao@cqu.edu.cn; 2 Key Laboratory of Technology for Construction of Cities in Mountain Area of Ministry of Education, Chongqing University, Chongqing 400030, China; 3 School of Public Health, University of Washington, Seattle, WA 98105, USA; MichaelAdeney0723@outlook.com

**Keywords:** hospital, function, COVID-19, outdoor space, resilience, safety, healthcare facilities, design, built environment

## Abstract

Hospital outdoor spaces play an important role for the safety and well-being of users (patients, visitors, and staff), particularly during a pandemic. However, the actual needs of these spaces are often overlooked due to the design and management process. This study investigates the perceptions of the public and occupants on the functional settings of outdoor spaces, and provides evidence for building a safe and resilient hospital during (and after) COVID-19. A multi-method approach of web content analysis (WCA) and a web-based survey was employed. Reports were collected from three mainstream websites; keywords were extracted and then categorized, pertaining to the functional settings of outdoor spaces. Three groups of occupants from Southwest Hospital (staff n = 47, patients n = 64, visitors n = 73) participated in the survey to identify their perceptions of these functional settings. Based on the 657 reports and 33 keywords selected, 7 functional settings were identified: health check (HC), quarantine and observation (QO), food and delivery (FD), healing and restoration (HR), waiting and rest (WR), transportation and parking (TP), load and unload (LU). From all users, HC (4.13) was thought to be the most expected function setting while FD (2.61) was the least. Regarding the satisfaction level, most users were satisfied with HC (3.22) while WR (2.16) was the least satisfying. The users also showed significant differences regarding expectation and satisfaction pertaining to their groups. The results indicate that the current outdoor space could not fully meet the needs of users, regarding the emerging functional setting, due to the pandemic. Users showed significant different perceptions on the functional setting due to their roles. The mismatch between the outdoor space and the users’ needs on emerging functional settings resulted in low satisfaction and high expectation in the survey. Environmental interventions with adaptive and flexible strategies should be adapted for these functional settings. The differences of users should be fully recognized by administrators, decision-makers, and designers.

## 1. Introduction

Since the outbreak of COVID-19, the built environment and urban environment have been profoundly disrupted and transformed due to the fear of infection, as well as to the emergent practices of lockdowns and social distancing [1]. Hospitals, as one of the most important built assets in the pandemic, are a collection of healthcare facilities—a part of an “anti-disease” infrastructure, providing a safe and resilient environment. During the pandemic, user expectations concerning the role of hospital outdoor spaces have risen dramatically, due to the perceived contributions to the safety and resiliency of hospitals. Disease transmission could be mediated by the physical environment of hospitals, including outdoor and indoor spaces, and a well-designed environment could make hospitals safer places for occupants [2]. Studies have buttressed the benefits of outdoor spaces, connecting them to the comfort, safety, and well-being of occupants in healthcare settings [3]. However, hospital outdoor spaces are often neglected due to the design and management process. In a hospital setting, the contributions of outdoor spaces, in regard to safety and resilience, are gradually being recognized [4].

Outdoor spaces could contribute toward controlling the spread of pathogens by providing places for health checks, quarantining, and medical resource reserves [5]. Observing the experiences in Wuhan, where COVID-19 first broke out, outdoor spaces could have assisted with the shortage of beds, treatment rooms, and heavy traffic at the very beginning of the crisis, becoming a precondition for determining the locations of cabin hospitals [6]. In temporary hospitals, reserving plenty of outdoor space is helpful for accommodating medical and supportive functions [7,8]. Outdoor space has also become a “buffer zone” between the hospital and nearby communities, preventing spread of the infection. This pandemic highlighted the role of outdoor spaces, indicating the growing demand for a better understanding of the functions and compositions of outdoor spaces.

From the perspective of a built environment, an outdoor space consists of spatial elements, such as entries to gardens, layouts, pathways, seating, planting, maintenance, amenities, etc. [9]. It is associated with the efficiency and safety of a hospital by accommodating different “flow” types, such as motor vehicles and pedestrians, staff and patients, and the “clean” and “contaminated” [10]. According to the building code in China (JB110-2008), outdoor space could account for as much as 70% of an area of a hospital site, with at least 30% greenery, indicating the aesthetic, restorative, and recreational value for a hospital [7]. Outdoor space is critical for achieving improved sustainability and the microclimate quality of a hospital, which affect the comfort and health of occupants [11]. Due to the high proportion of greenery, outdoor space could cool down a building and increase the comfort levels for patients [12]. It could serve as the urban public space between hospitals and neighborhoods [13]. Nonetheless, for a long time, the value and potential of outdoor spaces contributing toward safe and resilient environments have been underestimated.

As medicine has transformed (and is transforming), from a biological mode to an environmental–psychological–social mode [14], an increasing body of evidence suggests that outdoor spaces could enrich the health of hospital occupants, due to the restorative values of outdoor spaces [9,15]. Incorporating with natural components is proven to create a healing and restorative environment in a hospital [16]. Outdoor spaces could exert multiple benefits to the health of occupants, such as reducing stress and anxiety, supporting patient recovery, and addressing the emotional and social needs of a patient [15]. The greenery in outdoor space was associated with positive treatment outcomes, as well as the satisfaction level of patients from different ages [3,17]. Outdoor spaces could also produce comprehensive health benefits by providing places for physical activity and social interactions [18,19]. Although the hospital administrators are gradually realizing the benefits of outdoor spaces, the practice of utilizing outdoor space involves a “top-to-down” process, with little priority placed on the actual needs of hospital occupants, in particular, of the staff and visitors [20]. One reason is that the potential of outdoor space to accommodate emerging functions has not been fully explored, especially in developing countries. The evidence regarding the functional settings of outdoor spaces and user needs is limited and cannot fully support the practice of design and management.

In the midst of the COVID-19 pandemic, outdoor space can be converted and utilized as temporary areas to accommodate medical functions [21]. The provisions around urgent care buildings, in-patient departments, and offices make it possible to host rapidly constructed medical structures, such as tents, modular units, and other tensile solutions [22]. Some outdoor fields and venues were converted to temporary medical facilities, such as Mount Sinai Health System, NY, U.S., and Fangcang Shelter Hospital, Wuhan, China [22]. Most studies revealed that staying outside was safer because the virus was easily transmitted indoors than outdoors [23]. The practice of social distancing also encourages activities to be transferred from indoors to outdoors. Breathing fresh air outside, especially in a green space, seems to help reduce one’s chances of getting infected [24]. Well-designed and accessible outdoor spaces at hospitals are especially demanding in developing countries because of the restorative effects and medical functions [4]. Spacious outdoor spaces were thought to be a necessary condition for designated hospitals in COVID-19 because they could enable the rapid arrival and evacuation of vehicles, and installation of tents and medical facilities [22]. The entrances of hospitals were usually converted into health check sites, with body temperature monitors, waiting areas, and health code checkpoints. Nonetheless, knowledge concerning the functional settings of outdoor spaces is limited, resulting in utilization problems due to COVID-19. Temporary health checkpoints of outpatient services and emergencies were usually overcrowded, leading to a high risk of infection and low satisfaction of users. In some cases, temporary medical facilities and tents were difficult to construct in outdoor spaces due to insufficient area and lack of power and drainage [22]. Nonetheless, users were prone to stay outside due to the fear of indoor infection, but there was inadequate space for rest [25]. The pandemic has fundamentally changed the role of hospital outdoor space, and emerging functions need to be considered in the design, maintenance, and management of outdoor spaces. Previous studies have mainly focused on the indoor spaces of hospitals [3,26] rather than outdoor. Thus, there is a knowledge gap in both the research and practice due to the lack of studies about emergent scenarios. The current functional settings of outdoor spaces do not consider the emergent functions and actual needs of users in the pandemic, because their potential for building safe and resilient hospitals is always ignored. Most of the practice is based on the experiences, codes, and other official guidelines, but not the actual needs of occupants. Therefore, there is a need to explore the functional setting and user needs of outdoor spaces during the pandemic.

COVID-19 persists; it has exerted some permanent influence on certain aspects of hospitals and their users. This article explores the functional settings of outdoor spaces and the users’ actual needs during COVID-19. In contrast with previous studies, this study mainly collected evidence of functional settings from the public and occupants, through web content analysis (WCA) and a web-based survey. It provides evidence for building a safe and resilient hospital in the post COVID-19 era.

## 2. Methods and Materials

**Figure 1:**
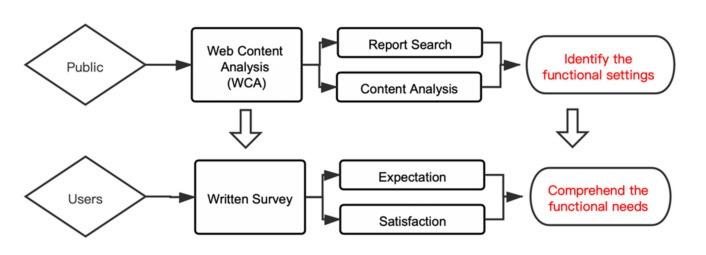
Research Framework.

We conducted multi-method research, combining WCA [27] and a web-based survey, to investigate the perceptions on functional settings from the public and hospital occupants, respectively (Figure 1). As an instructive approach, the survey provided static, intuitive, direct data with high value density; its sample size and authenticity were limited [28]. Contrarily, the WCA provided non-instructive data, from a large sample size, dynamic, real (but low value) density [29]. The combination of two methods allowed for comprehensive understanding of the topic and drew reliable conclusions by balancing their pros and cons. 

### 2.1. Instruments

#### 2.1.1. Web Content Analysis

In this study, WCA included two steps: web report searches and content analysis. It evolved from content analysis, which is a quantitative analysis based on qualitative data, to convert relevant reports into data, in quantities [29].

First, we applied the triangle combinations of keywords to search the reports on mainstream media websites (Table 1), using LocoySpider v9.4 (Lewei Information Technology Co., Ltd., China), a web crawler software, which is commonly adopted for web data mining. The information collected could reflect the real opinions from the public, indicating the needs and challenges of outdoor spaces. In most cases, it would not only provide sentiment and opinions from the public, but also serve as a channel to interact with the healthcare authority [30]. The collected reports were put into a dataset for the second screening. The inclusion criteria included: (1) it should be relevant to the functions of outdoor space; (2) it should focus on the functional issues in a hospital setting; (3) it should focus on the physical environment of outdoor space in accordance with the functions. Invalid data, such as repeated reports, unrelated news, and meaningless comments, were screened out.

Second, we employed ROSTCM v6.0 (Wuhan University, China) to conduct a content analysis on the reports. It could perform text segmentation and word frequency statistics for the texts on forums, websites, and blogs [31,32]. The reports were segmented and ranked in order to count the word frequency. The selected words were classified from the text segmentation and ranked from high to low, and then they were screened and identified by setting the frequency threshold (n > 30). The left words were processed by excluding the meaningless and ambiguous words, and then sorted into several main categories of functional settings, according to the intrinsic semantic relationship between the high-frequency words and manual interpretation, such as the logic of primary–secondary, cause–effect, and subordination.

#### 2.1.2. Web-Based Survey

The research team developed the online questionnaires, and distributed them to the respondents from staff, patients, and visitors in a hospital. This approach was adequate for this study—it was not only fast, low-cost, and contained fewer errors, it also met the “touchless” requirements due to COVID-19 measurements. The questionnaires were designed with three sections: (1) demographic information; (2) expectation on the functional setting; (3) satisfaction with the functional setting. Expectation and satisfaction were rated by the Likert scale (1–5). The demographic information included group, gender, age, and education. Expectation was used to estimate the perceived importance and necessity on the functional setting from respondents. Satisfaction was used to measure how respondents felt satisfied with the functional setting in the current outdoor space. At the end of the survey, respondents were asked to provide recommendations to optimize the outdoor space and any feedback they wished to share, if applicable. The online questionnaires were developed by the app “Questionnaire Star” (https://www.wjx.cn) on the WeChat platform, which was the most commonly used social media in China. The questionnaires were designed based on WCA results, and reviewed by experts to avoid violation of personal privacy. The reliability and validity of questionnaires were tested with Cronbach’s coefficients (0.771), KMO, and Bartlett test (0.754.), which proved suitable for the study. 

### 2.2. Data Source and Collection

Regarding WCA, three websites were selected as targets of report collections: People.com.cn, Xinhuanet.com, China.com.cn (4 June 2020). They were the top news websites regarding internet traffic in China, according to Alexa (https://alexa.chinaz.com 4 June 2020) (Table A1). Moreover, they were open access and the primary news gateways due to their relationships with the “authority”. The reports on these websites were all from official sources and updated timely with decent credibility and comprehensiveness. The search scope covered the reports from January to July 2020—the most emergent period for hospitals due to COVID-19. Three researchers worked on searching the reports and screened them from July to August 2020. Two more experts performed a content analysis of these reports and extracted keywords to identify the functional settings, from August to November 2020.

**Figure 2:**
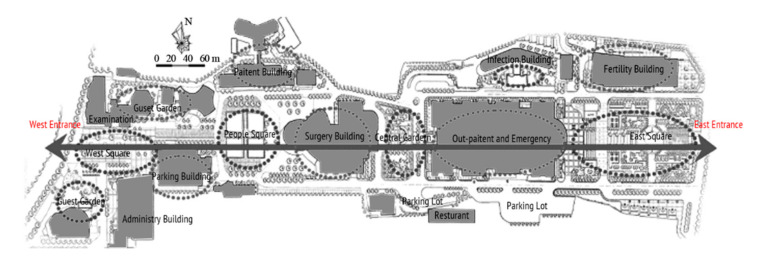
Site plan of Southwest Hospital.

A total of 47 staff members, 63 patients, and 73 visitors from Southwest Hospital, Chongqing, were selected as participants in this study. This hospital is one of the largest general hospitals in the southwest of China (Figure 2), which is typical for its layout and functional outdoor space settings. Moreover, it played an important role in curbing COVID-19 and faced many outdoor space problems for its location in the densely urbanized area. The vice president was contacted by a network of researchers to recruit staff for the survey. Their patients were also reached and invited to the survey once obtaining consent. Regarding the visitors, with the support of the hospital, our investigators distributed the questionnaires to them in the hospital. The questionnaires were distributed to participants through WeChat, so that they could fill them out without physical contact with others (Table A2). The survey lasted for a week in November 2020.

### 2.3. Statistical Analysis

In exception for the descriptive analysis—one-way ANOVA was performed within the different groups of users to compare their scores on satisfaction and expectations. Statistical analysis was conducted on SPSS V26.0 (IBM Corp). The significance level was set at *p* = 0.05.

## 3. Results

### 3.1. Functional Setting of Outdoor Space in the Hospital during COVID-19

**Figure 3:**
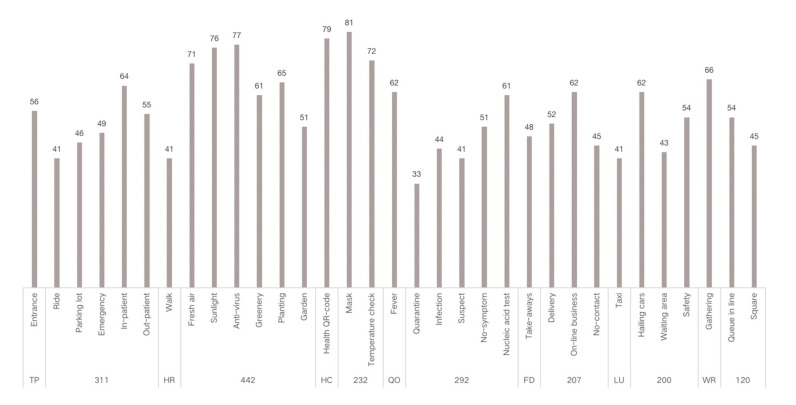
Frequency of keywords.

A total of 657 reports from 3 websites were obtained through WCA, and 78 high frequency words were extracted in the primary stage. After further screening and analyzing, 33 keywords of functions were identified and classified into 7 categories of functional setting (Figure 3). From the word frequency distribution and content clusters, the keywords of functions were distributed around topics of virus prevention, emergency response, waiting and resting, transportation, health restoration, and touchless practice. The top three frequency words were facemask (n = 81), antivirus (n = 77), and health QR code (n = 79), indicating the influence of the pandemic on the outdoor space.

The classified functional setting included health check (HC), quarantine and observation (QO), food and delivery (FD), healing and restoration (HR), transportation and parking (TP), waiting and rest (WR), and load and unload (LU) (Table 2). HC consisted of 3 keywords, with a total frequency count of 232, including testing body temperature, checking health code, or nucleic acid test report, which were mandatory when entering the hospital during the pandemic. QO consisted of three sub-functions with a total frequency (n = 292). It referred to the setting to accommodate suspected and confirmed cases temporally outside of the buildings. In an emergency, it could be converted into a temporary medical space to treat infected patients. FD (n = 207) consisted of four sub-functions, defined as functions for picking-up and dropping-off deliveries and food. The demand of FD surged during the pandemic and, accordingly, it was granted an independent category. HR (n = 442) consisted of seven sub-functions and was often described as the gardens for patients to recover and rest. It was considered the most important part of outdoor space and was granted an independent category. TP (n = 311) consisted of six sub-functions and mainly referred to the roads, parking lot, and walkways. It generally accounted for a large part of outdoor space in a hospital. WR (n = 120) consisted of three sub-functions, described as places for visitors to wait outside. WR was granted an independent category because people were encouraged to stay outside during the pandemic. LU (n = 200) referred to the functions of loading and unloading people when they came to (and left) the hospital; it consisted of four sub-functions. Since the transportation system in the hospital became more complicated than before, LU was supposed to accommodate all inbound and outbound “flows”, such as taxis, private cars, public transit, and hailing cars. In Southwest Hospital, all seven functional settings could be found along the east-to-west oriented axis, consisting of the East Square, Central Garden, Surgery Building, People Square, and West Square (Figure 2).

### 3.2. Demographic Information

Most participants were between 20 and 60 years old; there were more females than males (Table 3). More than half of the participants had undergraduate education and above. The education levels of staff members were apparently higher than the other two groups, of which 63.83% had graduate degrees and above. The proportion of seniors was the highest in the group of patients (60.94%) while the lowest among visitors. Most of the staff (60.94%) were females while most of the visitors were males (56.16%). As for the “portraits” of the participants: staff members were mostly high-educated females; visitors were mostly young males; and patients were mostly seniors. 

### 3.3. User Expectations of the Functional Settings of Outdoor Spaces

**Figure 4:**
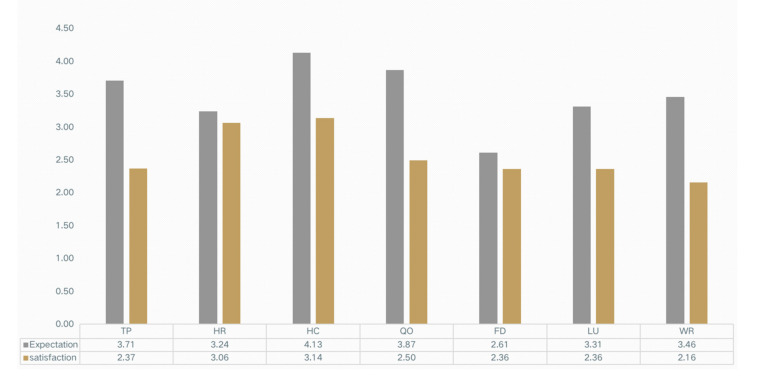
Comparison of the satisfaction and expectations of the functional setting.

**Figure 5:**
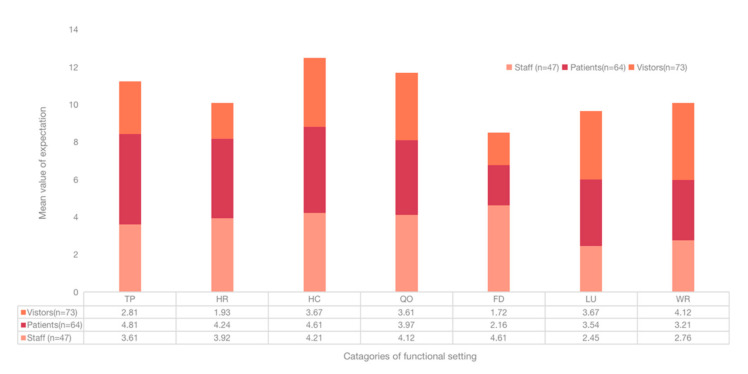
Expectation on the functional setting.

HC was rated as the highest mean value (4.13), whereas FD was rated lowest (2.61) (Figure 4), indicating that HC was thought to be the most important function setting. The mean value of QO (3.87) was the second highest, next to HC, revealing the impact of the pandemic to the outdoor space. Regarding the differences of groups (Figure 5), patients rated TP (4.81) as the highest and FD (2.16) the lowest. Visitors rated WR (4.12) the highest and FD (1.72) the lowers. Contrarily, staff rated FD (4.61) the highest and LU (2.45) the lowest, which indicates the differences on FD.

To further explore the relationship, one-way ANOVA was performed. It showed significant differences among FD (F = 21.53, *p* < 0.001), LU (F = −3.282. *p* = 0.002), and WR (F = −2.731, *p* = 0.007) from different groups (visitors, staff, patients) (Table 4). Meanwhile, the users from different ages (F = 2.679, *p* = 0.021) and education (F = 2.237, *p* = 0.041) showed significant differences on HR. Education could also affect user expectations on FD (F = 11.11, *p* < 0.001). The most significant differences were found in FD (2.89) and then HR (2.31) (Table 5). The results showed that different groups of participants had significant differences on the expectations of the functional setting.

### 3.4. Satisfaction of the Functional Setting

**Figure 6:**
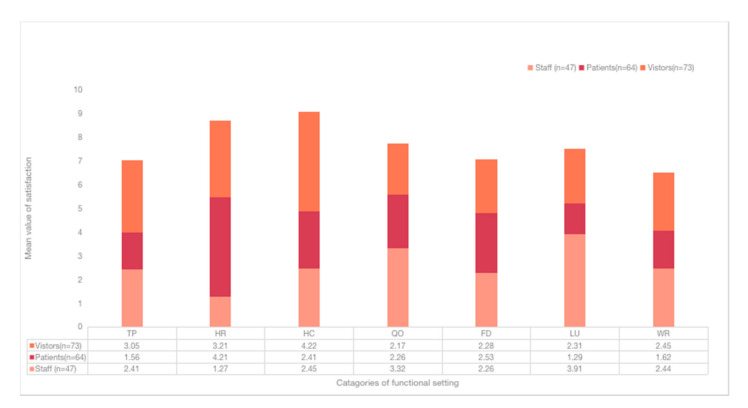
Satisfaction with the functional setting from the different groups.

Generally, the mean value of satisfaction was lower than the expectation of the functional setting (Figure 4), indicating the low levels of satisfaction of users on the current outdoor space. HC received the highest ratings (3.14), which was less than its counterpart, in expectation. WR received the lowest ratings (2.16), indicating the low levels of satisfaction from users. Regarding the differences of groups (Figure 6), patients rated HR (4.21) the highest, and LU (1.29) the lowest. The visitors rated HC (4.22) the highest and QO (2.17) the lowest. As for staff, they rated LU (3.91) the highest and HR (1.27) the lowest. The largest differences were found in HR (2.94) and LU (2.62), which were calculated from patients and visitors.

To further explore the relationship, one-way ANOVA was conducted and showed significant differences among HR (F = 2.956, *p* = 0.038), HC (F = 2.142, *p* = 0.034), and WR (F = −3.343, *p* = 0.003) from different groups (visitors, staff, patients) (Table 4). Participants from different education levels also showed significant differences on FD (F = 9.480, *p* = 0.009) and WR (F = −2.062, *p* = 0.041). The largest differences were found in HR (2.94) and LU (2.62) (Table 6). It indicated that groups of participants had different views on satisfaction in regard to the outdoor space. 

## 4. Discussion

### 4.1. Influence of the Pandemic and Outdoor Space in the Hospital

The pandemic posed a significant impact on the functional settings of outdoor spaces due to emerging medical needs and precautions. Users and the public both raised awareness about the value of outdoor spaces due to the fear of infection. Staying outdoors was usually thought to be safer and healthier than indoors during the pandemic. WCA results showed that more health and safety concerns emerged, and were incorporated into the functional settings of the outdoor space. Among the seven identified function settings, used had high expectations for HR, HC, and QO, indicating the awareness of health and safety associated with outdoor spaces. This pandemic promoted the role of outdoor spaces at hospitals because of the potential health benefits, such as sunshine, fresh air, and physical activity [33]. Outdoor space greenery could contribute toward a healing and restorative environment, which was proven to improve the mental and psychological health of occupants [18,19,34]. Patients could expect shorter recovery times and better mental statuses by having access to outdoor spaces with rich, natural features, both physically and visually [20,35]. The fatigue, stress, and medication errors of staff could also be alleviated by setting up green spaces around their working sites [36,37]. Outdoor space could also contribute toward the resilience of a hospital in coping with an emergency, by providing places to take care of unpredictable needs. These findings imply that health and safety will gradually become a major part of public perception on the use of outdoor space, influencing hospital research projects and practice in the future. In China, the authorities have already implemented health measures (e.g., regulations and building codes). For instance, it is mandatory to set up health checkpoints at the entrances of hospitals as well as fever clinics to screen suspected patients. This study shows the emergence of new functions, which came about from the pandemic; relevant measures of intervention should be considered, because the current layout could not fully meet these demands. Touchless practices and social distancing are accelerating the transformation of functional settings in outdoor spaces. Overall, the role that outdoor space plays at a hospital should be given adequate attention—during and after the pandemic.

### 4.2. Satisfaction with the Current Functional Setting of the Outdoor Space

The results showed that respondents were not very satisfied with the functional setting in the current outdoor space. The possible reason could be the mismatch between emerging functional needs and the current layout of the outdoor space. WR was rated the lowest by visitors on the satisfaction level because there was not much space available for it in the outdoor space, and environmental conditions were poor in this case. At Southwest Hospital, there were only two uncovered squares exclusively for WR, of which, the sizes were small and separated from the main building by roads. Visitors and patients had to wait around the building without shelter and seating, leading to traffic jams and a risk of contact. Patients rated LU the lowest (1.29) because the area for loading and unloading was insufficient, and different traffic flows often mingled; the distance to the entrance of hospital was often large. Visitors rated QO the lowest, indicating that there was not enough space for QO, which often caused conflicts. Staff were least satisfied with HR as there were few independent rest places available. Their current break areas were usually mixed with traffic flow; thus, they lacked privacy and safety. Patients rated high on HR (4.21), which was in contrast with the staff. At Southwest Hospital, there are several gardens for patients, but there is little consideration for staff. Since the outbreak of the virus, frontline staff members have faced much stress, and are lacking independent break areas to recover from fatigue. Moreover, due to assaulting incidents at the hospital, staff members need an independent outdoor area to heighten their sense of safety. Overall, occupants were not very satisfied with the outdoor space because it could not fully meet their actual needs of functions, due to the pandemic.

### 4.3. What Are the Expected Functional Settings of an Outdoor Space?

COVID-19-related functional settings received high expectations from the respondents, such as HC and QO, indicating user concerns about the risk of infection and increasing attention of health and safety. This encourages occupants to stay outdoors as much as possible to reduce the risk of infection, because it is almost impossible to practice social distancing and touchless measures indoors, especially in a crowded hospital. Patients rated TR the highest because traffic problems in the hospital were further aggravated by the pandemic. The precaution measures caused more waiting times and traffic delays, due to, e.g., body temperature checks, health code checks, etc. Furthermore, more people preferred to visit the hospital by private cars instead of public transit, as a means to avoid infection, causing a surging demand for parking lots. WR was granted expectations by visitors because they preferred to wait outside the building in the pandemic. During the pandemic, there were more procedures to enter a hospital building, especially for visitors. The concerns of being infected also encouraged visitors to remain outdoors. Staff placed expectations on FD, indicating the importance of delivery and other online services during the pandemic. It was not only a part of touchless practices, but it also helped save time for staff members, who were always busy in the hospital. To summarize, the pandemic exerted new challenges in regard to the use of outdoor space, and it is necessary to respond with appropriate functional settings to ensure the comfort, well-being, and safety of occupants.

### 4.4. The Role of Groups on the Respondents

Participants from different groups, ages, and educational backgrounds showed significant differences on their views of the functional settings of the outdoor space, which revealed a variation of their needs. Lack of a comfortable environment in the healthcare facilities was the main reason that hindered the satisfaction levels of occupants because their desires were not fully recognized [26]. It is important to address the different needs for functions, accordingly, in the hospital planning and management. The variables of groups played a more significant role in these differences than age and education. Understanding the needs from patients, visitors, and staff, respectively, has become essential. Regarding expectation, both patients and visitors rated FD the lowest because they rarely ordered delivery at the hospital. However, staff put much priority on FD, because ordering delivery and food could essentially save them time. As for satisfaction, patients were the least satisfied with LU, while staff rated it the highest. For patients, LU area were usually located at the main entrance, which was usually jammed with traffic and caused long wait times. For staff, they had a separate LU area, which was less affected by public flow. HR was the opposite—patients were quite satisfied with HR, whereas staff members were not. One possible reason is that patients usually have their own gardens or places for rest, whereas there are not enough of such places for staff members at Southwest Hospital. Overall, the users from different groups showed obvious differences in regard to their views on the functional settings of outdoor spaces; it is necessary to consider specific needs with a more comprehensive approach. 

### 4.5. Advice to the Practice

During COVID-19, the functional requirements of the outdoor spaces of hospitals are becoming more complex, whereas it is difficult for the current layouts to meet the emerging challenges. It is necessary to design and manage the outdoor space in an adaptive and flexible way. Flexibility means the capacity to improve the satisfaction level and sense of safety of different occupants in the hospital [20]. To achieve flexibility, the needs of different groups should be considered in the design and management, i.e., by setting up appropriate functional areas. According to the findings, for visitors, the WR area should be located around the outpatient and emergency buildings, and regarded as an extension of the indoor area, with easy access and a transitional space, so that the visitors would not be disrupted by poor weather while engaging outside. Moreover, the qualities of the WR area should be improved by providing facilities and amenities, such as a rest room, water fountain, and covered seating, which allow visitors to stay outside with comfort [38]. The way-finding system should be improved for easy navigation [33], and all WR areas should be connected by a paved pathway. Second, as for the patients, the TP area should be promoted, since patients are the least satisfied with this functional setting (even if it is the most expected functional setting by patients). The TP area should be connected to the LU area, with easy access, which allows for rapid inbound and outbound flow [39]. TP areas should be enlarged and separated from the walking entrances, since an increasing number of patients choose to visit hospitals by private cars. Hospitals should design welcoming and inviting garden entrances to separate the flow [40], which make patients aware of the PR and LU area. As for the staff, creating an independent HR area could be essential, as their stress and fatigue could be alleviated by having rest in the HR area, such as outdoor garden break areas. Natural features of outdoor spaces play an important role in benefiting psychological health, by helping people cope with negative emotions from the pandemic [41,42]. The HR area should be designed with close proximity to the work areas [36]. To provide views of the HR garden from inside the building would also encourage the use. The FD area should also be considered for staff, since staff members were the least satisfied with this functional setting (even if it is the most expected functional setting by staff). The designated area for picking up deliveries should be located close to their work place, and separated from the main entrance. 

It is necessary to improve the adaptability of the outdoor space, to transform from “normal” times to an emergency within the functional setting. Adaptability should become a fundamental part of the practice of an outdoor space, in order to ensure the reliability and efficiency of a hospital. First, multi-functional areas should be placed in an independent category in the zoning of outdoor spaces. Such areas could be either grassland or hard-covered, and need to be reserved to prepare for any emergent functional setting, such as HC and QO. The locations of such areas should be kept at a distance from the main hospital buildings, but having easy access to the main entrance. In China, many hospitals have set up fever clinics adjacent to the outpatient building to screen suspected cases, which proved to be effective at reducing the spread of COVID-19 indoors. According to the experiences in Wuhan, the ground of HC and QO areas should be pre-constructed with outlets of electricity, water, and drainage, to prepare for emergent functions. Second, the layout of the outdoor space should be organized with hierarchy and a system. For instance, clusters and fragments of outdoor spaces should be networked and connected with routes, allowing for the transformation of functional settings and the transition of different flows. In some new proposed hospital projects, the programs for both normal and emergent times are required in the planning and design. Third, since greenery accounts for a large part of outdoor spaces, its potential as an open space should be explored to allow for the engagement of occupants. By setting up seating and pathways, some greenery areas could be used for multiple purposes, for other functional settings, such as WR and HR, because the natural features of these areas could help “engage” users and stimulate their senses [39]. Overall, due to the uncertainty and complexity caused by the pandemic, the codes and guidelines of outdoors spaces should be updated in a flexible and adaptable way, in order to cope with the challenges from all scenarios.

### 4.6. Limitation

The sample size in this study was not large enough. In exception for the hospital setting, other types of healthcare facilities were not selected and investigated. As a result, it is still unclear whether the findings can be generalized to other healthcare facilities. It is assumed that no single blueprint fits all. Studies that are more empirical are needed to support the practice and evidence-based design in the post COVID-19 era. 

## 5. Conclusions

The functions of hospital outdoor spaces have been profoundly transformed by COVID-19, due to measures taken as a result of fear of the virus, infections, and lockdowns. Outdoor spaces are gaining increasing importance in the building of resilient and safe hospitals (i.e., against unpredictable challenges). To comprehend the actual needs from the public and hospital users, the study applied web content analysis and a web-based survey to explore their perceptions on the functional settings of outdoor spaces, divided into seven categorizes: health check (HC), quarantine and observation (QO), food and delivery (FD), healing and restoration (HR), waiting and rest (WR), transportation and parking (TP), and load and unload (LU). The functional settings of HC, QO, and HR are receiving attention from both the public and users due to the pandemic. HC was found to be the most “expecting” function in an outdoor space, while WR was the least satisfying. Users from different groups revealed diverse demands on the functional settings, regarding expectation and satisfaction. However, the current layout of outdoor space could not fully meet these functional requirements, and it is important to respond with a flexible and adaptive strategy. Practical advice is presented from two aspects. The findings could contribute to the research on outdoor spaces and the well-being of occupants in hospitals. This research outlines the gap among the public, users, and healthcare administrators in regard to outdoor spaces. This study highlights the necessity to incorporate the actual needs of users into the decision-making process (i.e., design and management). In the post COVID-19 era, these findings could serve as an inspiration and supportive evidence for creating safe and resilient environments.

## Figures and Tables

**Table 1:** Triangle Combinations of Keywords for Web Search.

Context	Architecture Setting	Study Body
Pandemic COVID-19 Virus	Hospital General hospital Infection disease hospital Temporary hospital	Outdoor space External environment Surrounding environment

**Table 2:** Functional settings of outdoor spaces.

Main Categories of Functional Settings	Sub-Categories of Functional Settings	Description	The Current Situations (Exampled at the Southwest Hospital)	Problems (Examples at the Southwest Hospital)
Health Check (HC)	Health QR-code Face mask Temperature check	To test body temperature, to check health QR code and nucleic acid test reports before entering the buildings.	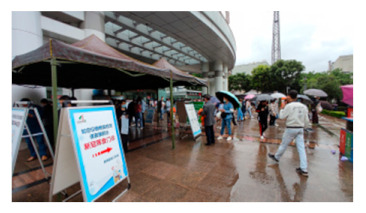	Crowded with different flows. Not enough space. Causing chaos in the entrance and exit.
Quarantine and Observation (QO)	Fever Quarantine Infection Suspect Non-symptom Nucleic acid test	To accommodate suspected fever patients and provide temporary medications.	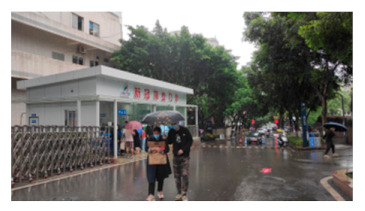	Difficult to find. Far from the main out-patient and emergency buildings. No shelters for the waiting crowds.
Food and Delivery (FD)	Take-away Delivery On-line business Touchless practice	To pick-up and drop-off deliveries and food.	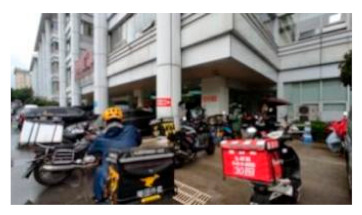	No designated area for delivery and foods. Chaos by the delivery, mingling with other “flows”. Delivery blocks the entrance of the building.
Healing and Restoration (HR)	Walk Fresh air Sunlight Greenery Anti-virus	To provide places and resources for patients and staff to recover, relax, and rest.	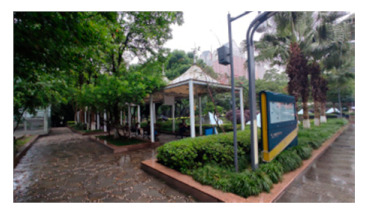	Far from the in-patient and staff buildings. No covered corridor connecting to the building. No smoking prohibited and low environmental qualities.
Transportation and Parking (TP)	Entrance Ride Emergency Parking lot In-patient Garden	To provide places and space for transportation and flow.	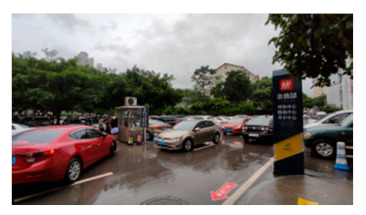	Not enough parking lot space. Chaos of motor flow mingling with pedestrians. Weak connection between public transit and entrance.
Waiting and Rest (WR)	Gathering Waiting in queue Square Outpatient	To provide places and space for visitors and patients to rest, gather, and wait.	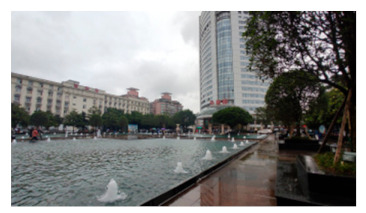	Not enough space for waiting crowds, but large water pool. No shelters in the waiting areas. No buffer zone between waiting area and roads.
Load and Unload (LU)	Taxi Hailing cars Waiting area Safety	To provide a place to load and unload people.	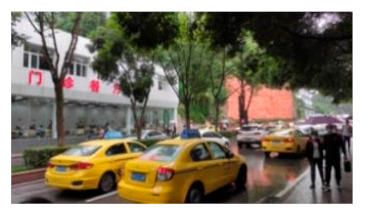	No designated area for loading and unloading. Chaos of flow, regarding taxis, hailing cars, private cars, and public transit.

**Table 3:** Survey demographic information.

		Staff		Patients		Visitors		Total	
		n	(%)	n	(%)	n	(%)	n	(%)
Age	<20	0	0.00%	2	3.13%	8	10.96%	10	5.43%
	20–40	23	48.94%	6	9.38%	38	52.05%	67	36.41%
	40–60	20	42.55%	17	26.56%	25	34.25%	62	33.70%
	>60	4	8.51%	39	60.94%	2	2.74%	45	24.46%
Gender	Male	12	25.53%	29	45.31%	41	56.16%	82	44.57%
	Female	35	74.47%	35	54.69%	32	43.84%	102	55.43%
Education	Junior college and below	0	0.00%	31	48.44%	41	56.16%	72	39.13%
	Undergraduate	17	36.17%	28	43.75%	29	39.73%	74	40.22%
	Graduate and above	30	63.83%	5	7.81%	3	4.11%	38	20.65%

**Table 4:** Expectation and satisfaction of the functional setting by demographic variables.

Variables		TP		HR		HC		QO		FD		LU		WR	
		T/F	*p*	T/F	*p*	T/F	*p*	T/F	*p*	T/F	*p*	T/F	*p*	T/F	*p*
Groups	Expectation	0.237	0.813	−0.946	0.346	0.066	0.936	1.712	0.636	**21.53**	**0.000**	**−3.282**	**0.002**	**−2.731**	**0.007**
	Satisfaction	0.332	0.741	**2.956**	**0.038**	**2.142**	**0.034**	1.155	0.283	−1.761	0.081	0.166	0.136	**−3.343**	**0.003**
Age	Expectation	0.215	0.832	**2.679**	**0.021**	0.069	0.945	1.921	0.165	0.472	0.639	0.084	0.892	0.021	1.839
	Satisfaction	0.806	0.421	−1.084	0.281	1.195	0.234	0.914	0.639	0.906	0.311	1.341	0.163	0.713	0.435
Gender	Expectation	0.753	0.453	−1.003	0.318	0.17	0.866	0.221	0.639	0.453	0.753	1.097	0.146	0.235	0.641
	Satisfaction	−1.896	0.071	−0.295	0.769	0.554	0.581	4.714	0.095	−2.196	0.051	0.754	0.482	0.199	0.889
Education	Expectation	−0.651	0.516	**2.237**	**0.041**	1.276	0.226	0.512	0.478	**11.110**	**0.000**	1.121	0.321	1.655	0.201
	Satisfaction	1.539	0.219	1.245	0.297	−1.266	0.208	0.137	0.718	**9.480**	**0.009**	−1.415	0.178	**−2.062**	**0.041**

Sample size = 184; T = value for independent-samples *t*-test; F = value for ANOVA; Sig. is set at 0.05 level and value is bold. The approximate normal distributions and equal variances were tested and satisfied by the Shapiro–Wilk test of normality and Levene’s test for homogeneity.

**Table 5:** Differences on the expectations of the functional setting by different groups.

Indicators	Patients (Mean)	Visitors (Mean)	Staff (Mean)	Maximum	Minimum	Differences between Max and Min
TP	3.61	4.81	2.81	4.81	2.81	2.00
HR	3.92	4.24	1.93	4.24	1.93	2.31
HC	4.21	4.61	3.67	4.61	4.21	0.40
QO	4.12	3.97	3.61	4.12	3.61	0.51
FD	4.61	2.16	1.72	4.61	1.72	2.89
LU	2.45	3.54	3.67	3.67	2.45	1.22
WR	2.76	3.21	4.12	4.12	2.76	1.36

**Table 6:** Differences on the satisfaction with the functional setting by the different groups.

Indicators	Patients (Mean)	Visitors (Mean)	Staff (Mean)	Maximum	Minimum	Differences between Max and Min
TP	2.41	1.56	3.05	3.05	1.56	1.49
HR	1.27	4.21	3.21	4.21	1.27	2.94
HC	2.45	2.41	4.22	4.22	2.41	1.81
QO	3.32	2.26	2.17	3.32	2.17	1.15
FD	2.26	2.53	2.28	2.53	2.26	0.27
LU	3.91	1.29	2.31	3.91	1.29	2.62
WR	2.44	1.62	2.45	2.45	1.62	0.83

## Data Availability

The data could be available upon reasonable requirements through corresponding author.

## Appendix A. Traffic Statistics of Three Selected Websites

**Table A1:** Traffic Statistics of Three Selected Websites.

	Global Rank	Chinese Rank	Gateway Site Rank	Daily Traffic
People.com.cn	626	22	6	2,478,214
China.com.cn	19,238	194	12	103,179
Xinhuanet.com	16	92	9	149,126,786

Note: all data are sourced from: https://top.chinaz.com; the data were recorded on 4 June 2020.

## Appendix B. Questionnaire for Respondents’ Expectations and Satisfaction of the Functional Settings of the Outdoor Space at the Southwest Hospital

**Table A2:** Questionnaire for Respondents’ Expectations and Satisfaction of the Functional Settings of the Outdoor Space at the Southwest Hospital.

Individual Information					
**Role**	**Patient**	**Visitor**	**Staff**		
Age					
Gender					
Education Level	Junior college and below	Undergraduate	Master’s degree and above		
Expectations on the seven functional settings of outdoor space at the hospital					
Instruction: how do you feel about the importance and necessity of the seven functional settings in the outdoor space, during and after COVID-19? Please rate from 1–5; 1 (not necessary or important), 5 (very necessary or important).					
	1	2	3	4	5
Health check (HC)					
Quarantine and observation (QO)					
Food and delivery (FD)					
Healing and restoration (HR)					
Transportation and parking (TP)					
Waiting and rest (WR)					
Load and unload (LU)					
Satisfaction on the seven functional settings of the outdoor space at the hospital					
Instruction: do you feel satisfied with the seven functional settings currently in the outdoor space, during and after COVID-19? Please rate from 1–5; 1 (not satisfied at all), 5 (very satisfied).					
	1	2	3	4	5
Health check (HC)					
Quarantine and observation (QO)					
Food and delivery (FD)					
Healing and restoration (HR)					
Transportation and parking (TP)					
Waiting and rest (WR)					
Load and unload (LU)					
Any recommendations and feedback regarding the current outdoor space.					
